# BmK NSPK, a Potent Potassium Channel Inhibitor from Scorpion *Buthus martensii* Karsch, Promotes Neurite Outgrowth via NGF/TrkA Signaling Pathway

**DOI:** 10.3390/toxins13010033

**Published:** 2021-01-05

**Authors:** Fang Zhao, Xiaohan Zou, Shaoheng Li, Jing He, Chuchu Xi, Qinglian Tang, Yujing Wang, Zhengyu Cao

**Affiliations:** Department of TCM Pharmacology, School of Traditional Chinese Pharmacy, China Pharmaceutical University, Nanjing 211198, China; zhaofang0927@cpu.edu.cn (F.Z.); imzouxiaohan@cpu.edu.cn (X.Z.); lishaoheng0402@stu.cpu.edu.cn (S.L.); Hejing0902@stu.cpu.edu.cn (J.H.); xichuchu0917@stu.cpu.edu.cn (C.X.); tangqinglian0729@stu.cpu.edu.cn (Q.T.)

**Keywords:** neurite outgrowth, scorpion toxin, potassium channel, nerve growth factor

## Abstract

Scorpion toxins represent a variety of tools to explore molecular mechanisms and cellular signaling pathways of many biological functions. These toxins are also promising lead compounds for developing treatments for many neurological diseases. In the current study, we purified a new scorpion toxin designated as BmK NSPK (*Buthus martensii* Karsch neurite-stimulating peptide targeting K_v_ channels) from the BmK venom. The primary structure was determined using Edman degradation. BmK NSPK directly inhibited outward K^+^ current without affecting sodium channel activities, depolarized membrane, and increased spontaneous calcium oscillation in spinal cord neurons (SCNs) at low nanomolar concentrations. BmK NSPK produced a nonmonotonic increase on the neurite extension that peaked at ~10 nM. Mechanistic studies demonstrated that BmK NSPK increased the release of nerve growth factor (NGF). The tyrosine kinases A (TrkA) receptor inhibitor, GW 441756, eliminated the BmK NSPK-induced neurite outgrowth. BmK NSPK also increased phosphorylation levels of protein kinase B (Akt) that is the downstream regulator of TrkA receptors. These data demonstrate that BmK NSPK is a new voltage-gated potassium (K_v_) channel inhibitor that augments neurite extension via NGF/TrkA signaling pathway. K_v_ channels may represent molecular targets to modulate SCN development and regeneration and to develop the treatments for spinal cord injury.

## 1. Introduction

Scorpion venoms are the biological weapon for scorpion to capture their prey and defend against their predators. Scorpion intoxication has been frequently reported worldwide, especially in Israel [1] and the United States of America [2]. The Chinese scorpion, *Buthus martensii* Karsch (BmK), is less toxic. However, as a Chinese traditional medicine, BmK has been widely used for thousands of years to treat facial palsy [3], epilepsy [4], stroke [5], and pain [6] in the clinical practice. Scorpion venoms contain hundreds of structurally diverse peptides with distinct biological activities. These peptides can modulate the activities of many plasma-membrane-tethered channels/receptors and the proteins expressed in the endoplasmic reticulum, often with high affinity and high selectivity [7]. Ion channels are the main targets of scorpion toxins. Studies have demonstrated that scorpion toxins can modulate the gating kinetics of many ion channels, including voltage-gated potassium channels (K_v_ channels) [8], voltage-gated sodium channels (VGSCs) [7,9], voltage-gated calcium channels (Ca_v_ channels) [10], chloride channels [11], and ryanodine receptors [12]. Due to their high affinity and selectivity, scorpion toxins are widely used as tools to explore the gating mechanism of ion channels [13] and their downstream cellular signaling pathways [14]. Typically, long-chain scorpion toxins (60–74 amino acid residues) bind to VGSCs, whereas the short-chain scorpion toxins (30–40 amino acid residues) modify K_v_ channel activity. All scorpion K_v_ channel inhibitors form a common cysteine-stabilized α-helix-β-sheet (Csαβ) motif [15].

Activity-dependent neurite outgrowth is a critical step to the proper neuronal network formation both in developing nerve system and during nerve regeneration [16]. Ca^2+^ signaling plays key roles in activity-dependent dendritic arborization, axonal elongation, and synapse formation [17]. Modulation of ion channel activity can directly or indirectly affect the Ca^2+^ signaling. For example, both VGSC channel gating modifiers and K_v_ channel blockers have been reported to alter the fidelity of the Ca^2+^ signaling and modulate the neurite outgrowth in cortical neurons [18,19]. Recently, several long-chain scorpion toxins were reported to display neurotropic effect both in in vivo model [20] and in primary cultured neurons [7,21] by increasing brain-derived neurotrophic factor (BDNF) expression and phosphorylation levels of cyclic adenosine monophosphate (cAMP)-response element binding (CREB) and extracellular-regulated protein kinases 1/2 (ERK1/2) proteins.

In the present study, we aimed to discover new scorpion peptide that can modify neuronal Ca^2+^ dynamics and to explore its potential in the stimulation of neurite outgrowth. We purified a new scorpion peptide designated to be BmK neurite-stimulating peptide targeting K_v_ channels (NSPK) from BmK venom. BmK NSPK potently increased spontaneous calcium oscillation (SCO) frequency and amplitude and inhibited outward potassium channel current without affecting the VGSC channel activity in spinal cord neurons (SCNs). BmK NSPK stimulated neurite outgrowth, increased the release of nerve growth factor (NGF) and the phosphorylation levels of protein kinase B (Akt) that is downstream signaling molecules of tyrosine kinases A (TrkA) receptors. Furthermore, TrkA receptor inhibitor, GW 441756 (GW), eliminated the BmK NSPK-induced neurite outgrowth, demonstrating that BmK NSPK, a new K_v_ inhibitor, enhanced neurite outgrowth through NGF/TrkA signaling pathway.

## 2. Results

### 2.1. BmK NSPK Purification and Structure Determination

**Figure 1:**
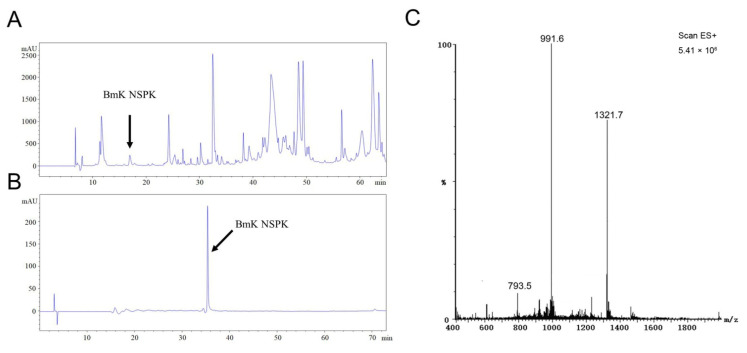
HPLC purification of *Buthus martensii* Karsch neurite-stimulating peptide targeting K_v_ channels (BmK NSPK). (**A**) Representative RP-HPLC chromatogram of the fraction eluted with 0.5 M NaCl in an CM-Sephadex C-50 cation ion-exchange column. The column was eluted with different percentages of solvent A (0.1% Trifluoroacetic acid (TFA) in ddH_2_O) and solvent B (0.085% TFA, 70% acetonitrile in ddH_2_O): from 0 to 5 min, 19% B, 5–20 min, 28% B; 20–55 min, 48% B; 55–60 min, 19% B at a flow rate of 2 mL/min. The arrowhead indicates the peak of BmK NSPK. (**B**) Representative RP-HPLC chromatogram of purified BmK NSPK eluted with gradient of acetonitrile: solvent A (0.1% TFA in ddH_2_O) and solvent B (0.085% TFA, 70% acetonitrile in ddH_2_O): from 0 to 10 min, 99.5% B; 10–70 min, 58% B; 70–75 min, 99.5% B at a flow rate of 1 mL/min. A single peak (indicated by arrowhead) was observed, suggesting that BmK NSPK is of high purity. (**C**) ESI-MS of BmK NSPK. The multiple ion charges of 1321.7, 991.6, and 793.5 m/z corresponded to [M+3H]^3+^, [M+4H]^4+^, and [M+5H]^5+^, respectively.

Scorpion toxins have been demonstrated to regulate the activities of a variety of ion channels by modulating their gating kinetics and therefore modulating neuronal activity [7,9,18,21]. We therefore further analyzed BmK venom components to discover new scorpion peptide that can modify neuronal Ca^2+^ dynamics and to explore its potential in the stimulation of neurite outgrowth, a critical step for the nerve development and nerve regeneration [22]. After size-exclusive ion-exchange and RP-HPLC chromatography, a single peak at retention time of 16.95 min designated to be BmK NSPK was collected (Figure 1A). The unitality of the peptide was accessed by RP-HPLC (Figure 1B). ESI-MS gave the multiple ion charges of 1321.7, 991.6, and 793.5 m/z that corresponded to [M+3H]^3+^, [M+4H]^4+^, and [M+5H]^5+^, respectively, demonstrating that the molecular weight of BmK NSPK was 3962.3 Da, which is distinct from the molecular weights of currently reported BmK toxins (Figure 1C).

**Figure 2:**
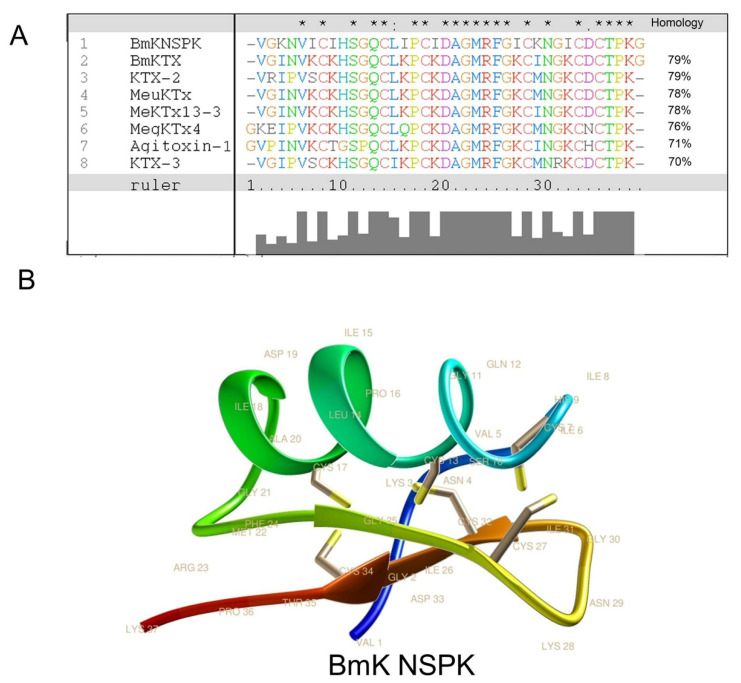
Homology of BmK NSPK with other scorpion toxins. (**A**) Multiple alignment analysis using ClustalX software showed that BmK NSPK displays over 70% similarity with reported voltage-gated potassium (K_v_) channel blockers. The asterisk, dot, and colon above the sequences represent the concordance of amino acid residues in the same position: the asterisk means 100% similarity, the dot means semiconservative mutation, and the colon means conservative mutation. (**B**) Three-dimensional (3D) structure modeling of BmK NSPK using *Buthus martensii* Kaliotoxin (BmKTX, PDB code: 1BKT) as a temperate. Yellow sticks indicate the disulfide bridges.

The primary amino acid sequence of BmK NSPK was determined to be VGKNVICIHSGQCLIPCIDAGMRFGICKNGICDCTPKG (Figure 2A) using Edman degradation with theoretic molecular weight (MW) of 3962.9 Da that is consistent with the molecular weight determined by ESI-MS. Analysis using Clustal X software showed that BmK NSPK displayed over 70% sequence homology to current known K_v_ channel blockers such as *Buthus martensii* Kaliotoxin (BmKTX) [23], Kaliotoxin 2 (KTX-2) [24], *Mesobuthus eupeus* potassium channel toxin (MeuKTx) [25], and *Mesobuthus eupeus* potassium channel toxin 13-3 (MeKTx13-3) [26] (Figure 2A). Homology modeling using BmKTX as a template [23] demonstrated that BmK NSPK belonged to classical Csαβ potassium channel blockers from scorpion venom [27] that contained one α-helix and two antiparallel β-sheets (Figure 2B). The α-helix of BmK NSPK peptide comprised residues Leu^14^ to Asp^19^, which was slightly shorter than the α-helix of BmKTX (Leu^14^ to Ala^20^) [23]. However, the difference of the α-helix folding between BmK NSPK and BmKTX was marginal. The two antiparallel β-sheets of BmK NSPK were from Gly^25^ to Cys^27^ and Ile^31^ to Cys^34^, similar to that of BmKTX [23].

### 2.2. BmK NSPK Altered Calcium Dynamics and Depolarized Membrane in Primary Cultured SCNs

**Figure 3:**
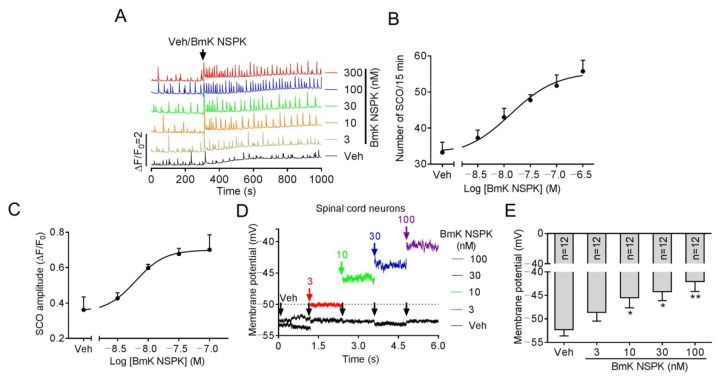
BmK NSPK-augmented spontaneous Ca^2+^ oscillations and depolarized membrane in spinal cord neurons (SCNs). (**A**) Representative traces showing BmK NSPK effect on spontaneous calcium oscillation (SCOs) in SCNs. (**B**) Concentration–response curve of BmK NSPK-altered SCO frequency. (**C**) Concentration–response curve of BmK NSPK-altered SCO amplitude. N = 4 wells. (**D**) Representative traces showing BmK NSPK effect in membrane potential (0 pA input) before and after addition of BmK NSPK in SCNs. The black arrowheads indicated the addition of vehicle control. The red, green, blue, and purple arrowheads indicated the additions of different concentrations of BmK NSPK. (**E**) BmK NSPK depolarizes SCNs membrane. *, *p* < 0.05, **, *p* < 0.01, vs. Vehicle (Veh).

The main targets of scorpion toxins are ion channels that orchestrate Ca^2+^ dynamics in neurons [7,21] that define the pattern of gene expression and neuronal network formation [28]. Primary cultured SCNs display synchronized spontaneous calcium oscillations (SCOs) [7] that are balanced by excitatory and inhibitory neuronal inputs [28,29]. Alteration of the pattern of SCOs has been used to discover the neuroprotective compound for seizure [30] and pain [31], and also to discover neurotoxicants and explore their mechanisms [18,32,33]. We therefore explored the influence of BmK NSPK on SCOs in primary cultured SCNs. Addition of BmK NSPK produced a rapid and sustained increase in both the amplitude and frequency of SCOs (Figure 3A) with the concentration of toxin at half-maximal efficacy (EC_50_) values of 13.4 nM (4.00–45.1 nM, 95% confidence intervals, 95% CI, Hill slope = 1.0) and 6.22 nM (2.22–17.4 nM, 95% CI, Hill slope = 1.0), respectively (Figure 3B,C). Application of BmK NSPK also resulted in a concentration-dependent membrane depolarization (Figure 3D,E).

### 2.3. BmK NSPK Directly Inhibits K_v_ Channels in Spinal Cord Neurons

**Figure 4:**
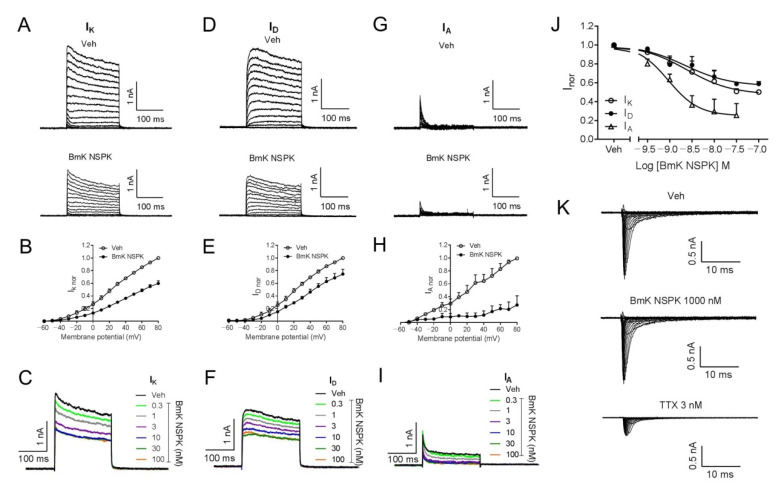
BmK NSPK directly inhibited outward K^+^ currents in SCNs and had no effect on the Na^+^ currents. (**A**) Representative traces for BmK NSPK inhibition of outward K^+^ (I_K_) currents (transient components (I_A)_ + sustained delayed-rectifier components (I_D)_) elicited by depolarizing potentials ranging from −60 to +80 mV (10 mV increase) from a holding potential of −110 mV. (**B**) Current–voltage (I-V) curve of 300 nM BmK NSPK inhibition of I_K_ currents. (**C**) Representative traces of I_K_ currents elicited by a step depolarization from a holding potential of −110 mV to +70 mV in the absence and presence of different concentrations of BmK NSPK. (**D**) Representative traces for BmK NSPK inhibition of I_D_ currents elicited by stepping to the potentials ranging from −60 to +80 mV (10 mV increment) from a pre-depolarized potential of −40 mV for 200 ms. (**E**) Current–voltage (I-V) curve of 300 nM BmK NSPK inhibition of I_D_ currents. (**F**) Representative traces of I_D_ currents elicited by a step depolarization from a pre-depolarized potential of −40 mV to +70 mV in the absence and presence of different concentrations of BmK NSPK. (**G**) Representative traces for BmK NSPK inhibition of I_A_ currents. I_A_ currents were obtained by subtracting I_D_ currents (**D**) from I_K_ currents (**A**). (**H**) Current–voltage (I-V) curve of 300 nM BmK NSPK inhibition of I_A_ currents. (**I**) Representative traces of I_A_ currents in the absence and presence of different concentrations of BmK NSPK. (**J**) Concentration–response curves of BmK NSPK inhibition of I_K_, I_D_, and I_A_ currents. (**K**) Representative traces of Na^+^ currents elicited by depolarizations from −100 mV to +45 mV in the absence and presence of BmK NSPK (1000 nM) or tetrodotoxin (TTX, 3 nM). TTX but not BmK NSPK inhibited Na^+^ currents in cultured SCNs. N = 4–5 neurons.

Short-chain scorpion toxins mainly affect K_v_ channel activity [24], although exceptions exist [34]. We therefore assessed BmK NSPK effect on the K_v_ channels in SCNs. In SCNs, outward K^+^ currents (I_K_) that contain both transient (I_A_) and sustained delayed-rectifier (I_D_) components are responsible for the repolarization of action potentials [35,36]. To examine the influence of BmK NSPK on K_v_ channels, the outward K^+^ currents (I_K_ = I_A_ + I_D_) were elicited by depolarizing potentials ranging from −60 to +80 mV (10 mV increase) from a holding potential of −110 mV (Figure 4A). Application of BmK NSPK (300 nM) inhibited the I_K_ currents at all depolarized potentials (Figure 4A,B). The inhibitory effect of BmK NSPK on the I_K_ currents was voltage-dependent (Figure 4B). BmK NSPK concentration-dependently suppressed the I_K_ currents elicited by a depolarization from −110 mV to +70 mV (Figure 4C) with IC_50_ value of 2.42 nM (0.68–8.61 nM, 95% CI, Hill slope = −0.86) (Figure 4J). To record the I_D_ currents, cells were pre-depolarized to a potential of −40 mV for 200 ms that inactivated I_A_ current, before stepping to the potentials ranging from −60 to +80 mV (10 mV increment) (Figure 4D). A concentration of 300 nM of BmK NSPK inhibited I_D_ currents elicited by a series of depolarization potentials with voltage dependence (Figure 4D,E). BmK NSPK concentration-dependently suppressed the I_D_ currents elicited by a depolarization from a holding potential of −40 mV to +70 mV (Figure 4F) with an IC_50_ value of 2.24 nM (0.68–7.44 nM, 95% CI, Hill slope = −0.86) (Figure 4J). To measure the I_A_ currents, I_D_ currents were subtracted from the total of the outward I_K_ currents at each respective depolarizing potential. BmK NSPK (300 nM) suppressed I_A_ currents at each depolarizing potential (Figure 4G,H). BmK NSPK concentration-dependently suppressed the I_A_ currents (Figure 4I) with an IC_50_ value of 0.93 nM (0.41–2.13 nM, 95% CI, Hill slope = −1.27) (Figure 4J). In contrast to K_v_ channels, BmK NSPK (1000 nM) had no effect on the Na^+^ currents elicited by depolarizing steps from a holding potential of −100 mV to +45 mV in a 5 mV increment, whereas tetrodotoxin (TTX, 3 nM) suppressed the Na^+^ currents (Figure 4K). The maximal inhibition by BmK NSPK of I_D_ currents was ~43%, whereas the maximal inhibition by BmK NSPK of I_A_ currents was much greater, reaching ~75% (Figure 4J).

### 2.4. BmK NSPK Enhances Neurite Outgrowth in Cultured SCNs

**Figure 5:**
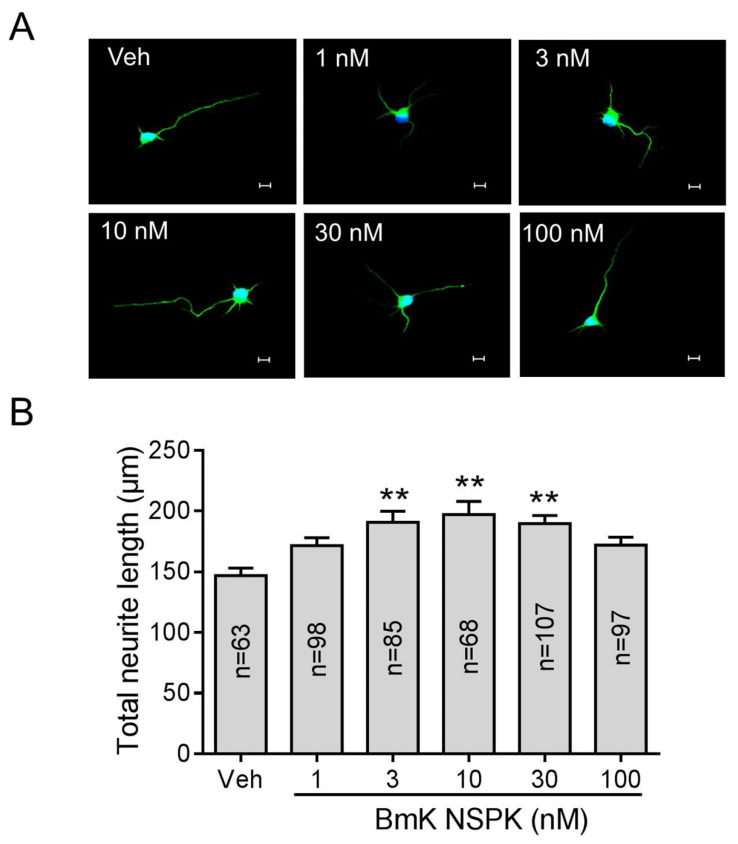
BmK NSPK enhanced neurite outgrowth. (**A**) Representative immunofluorescence images of SCNs stained with microtubule-associated protein-2 (MAP2) and Hoechst 33,342. Scale bar: 10 μm. (**B**) Quantification of BmK NSPK response on neurite extension. N means the number of neurons. **, *p* < 0.01, vs. Veh.

Small molecules inhibiting K_v_ channels are reported to augment SCOs and stimulate neurite outgrowth [18,37]. We therefore examined the ability of BmK NSPK to stimulate neurite outgrowth in SCNs. Application of BmK NSPK for 48 h commencing 5 h post-plating stimulated neurite extension in cultured SCNs in a nonmonotonic manner, with maximal stimulation occuring at the concentration around 10 nM (Figure 5A,B).

### 2.5. BmK NSPK-Induced Neurite Outgrowth is Dependent on NGF/TrkA Signaling Pathway

**Figure 6:**
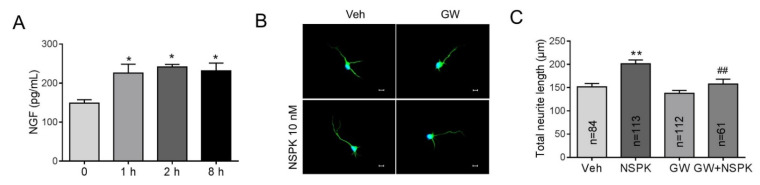
BmK NSPK increased nerve growth factor (NGF) release and enhanced neurite extension via tyrosine kinases A (TrkA) receptor. (**A**) 10 nM BmK NSPK increased NGF release. *, *p* < 0.05, BmK NSPK vs. Veh (n = 4). (**B**) Representative immunofluorescent pictures of SCNs stained with MAP2 and Hoechst 33,342 after Veh or BmK NSPK (10 nM), BmK NSPK + GW 441756 (GW, 1 µM) exposure for 48 h. Scale bar: 10 μm. (**C**) Quantification of GW effect on BmK NSPK-induced neurite extension. N means the number of neurons. **, *p* < 0.01, vs. Veh; ^##^, *p* < 0.01, vs. BmK NSPK.

NGF has been reported to enhance the neurite extension in rat adrenal pheochromocytoma (PC12) cells [38] and sensory neurons [39] via TrkA receptors. We therefore examined whether BmK NSPK can modulate the release of NGF in SCNs. Application of BmK NSPK (10 nM) produced a significant release (~50%) of NGF that lasted for at least 8 h (Figure 6A). To examine whether BmK NSPK-induced neurite outgrowth was though TrkA receptors, a specific TrkA receptor inhibitor, GW 441756 (GW), was added 30 min before BmK NSPK exposure. GW (1 μM) abolished BmK NSPK-induced neurite outgrowth (Figure 6B,C), suggesting that BmK NSPK promoted neurite outgrowth through NGF-induced TrkA signaling pathway.

**Figure 7:**
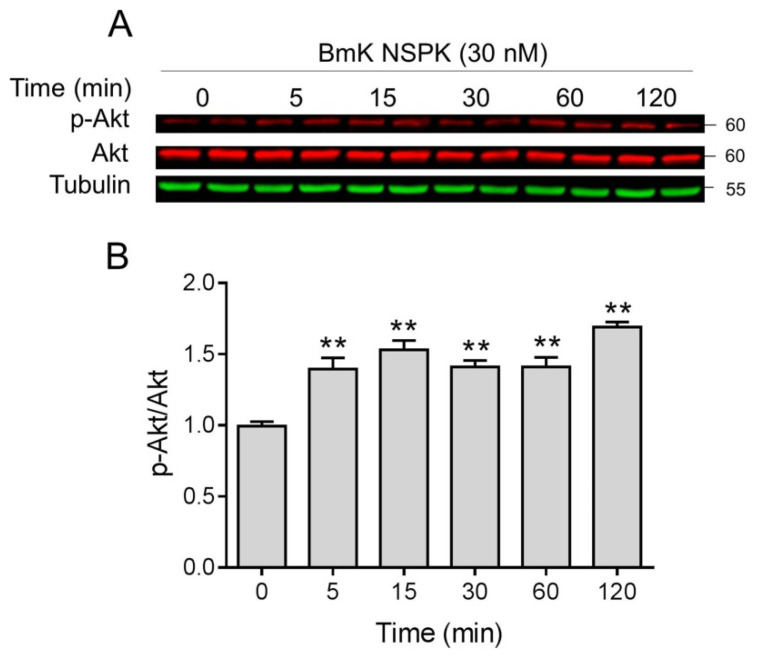
BmK NSPK phosphorylated protein kinase B (Akt) in primary SCNs. Representative western blots (**A**) and quantification (**B**) for BmK NSPK (30 nM)-stimulated phosphorylation of Akt. Each point represents mean ± SEM (n = 4). **, *p* < 0.01, vs. Veh.

NGF binding to TrkA receptors can activate downstream regulators such as Akt, which have been reported to contribute to neuronal differentiation and dendritic arborization [40,41]. We therefore examined whether Akt was activated after BmK NSPK exposure. BmK NSPK (30 nM) rapidly increased phosphorylated levels of Akt as early as 5 min after exposure, and this effect lasted at least for 30 min (Figure 7).

## 3. Discussions

Scorpion toxins are invaluable tools to explore the gating mechanism of many ion channels as well as the downstream cellular signaling pathways due to their high affinity and selectivity [42,43]. These toxins are also lead compounds in the development of treatments for many neurological diseases, such as pain [6]. In the current study, we purified a new short-chain scorpion peptide designated to be BmK NSPK. Structurally, BmK NSPK displayed over 70% sequence homology to current known K_v_ channel blockers such as BmKTX [23], KTX-2 [24], MeuKTx [25], and MeKTx13-3 [26]. However, difference in the primary sequence was significant between BmK NSPK and BmKTX. BmK NSPK contained many isoleucine residues (Ile^6^, Ile^8^, Ile^15^, Ile^18^, Ile^26^, and Ile^31^) that in BmKTX, KTX-2, MeuKTx, and MeKTx13-3 were conserved to be a lysine. Homology modeling using BmKTX as a template [23] demonstrated that BmK NSPK contained a short α-helix (Leu^14^–Asp^19^) connected by a tight turn to a two-stranded antiparallel β-sheet (Gly^25^–Cys^27^ and Ile^31^–Cys^34^), suggesting that BmK NSPK contained a classical Csαβ motif, a conserved three-dimensional structure of K_v_ channel blockers from different species of scorpion venoms [27].

Pharmacologically, BmK NSPK suppressed outward potassium channel currents with potency at single-digit nanomolar concentrations. It should be mentioned that although the relative potencies of BmK NSPK on the suppression of I_A_ and I_D_ currents were similar, the maximal inhibition of BmK NSPK on I_A_ currents was much greater than that on I_D_ currents, suggesting that transient K_v_ channels were likely more susceptible to BmK NSPK exposure. However, inhibition of I_K_, I_A_, and I_D_ currents by BmK NSPK were incomplete even at the highest concentration (100 nM). This is likely because some subtypes of I_D_ and I_A_ channels are not sensitive to BmK NSPK. The selectivity of BmK NSPK on subtype of K_v_ channels needs further exploration. BmK NSPK displayed similar potency to highly homologous scorpion toxins such as BmKTX [44], MeuKTX [25], and MeKTx13-2 [26], which suppressed K_v_ channels with potencies from high picomolar to low nanomolar concentrations. The amino acids of Arg^23^, Lys^26^, and Asn^29^ have been reported to be the key residues for BmKTX to suppress K_v_ channels [23]. Although amino acid residues (Arg^23^ and Asn^29^) were conserved between BmK NSPK and BmKTX, the residue (Lys^26^) in BmKTX was mutated to be an Ile^26^ in BmK NSPK. However, the affinities of BmK NSPK and BmKTX on K_v_ channels appeared to be similar.

It has been reported that universal K_v_ channels blocker as well as an array of specific K_v_1 subfamily inhibitors augment the SCO activity in cortical neuronal cultures [18]. Similarly, suppression of K_v_ channels with Ba^2+^ has been shown to augment SCO activity in hypothalamic neurons [45]. SCO profile in spinal cord neuronal cultures is dependent on the action potential generation and is orchestrated by both neuronal excitatory and inhibitory inputs [46]. The release of Ca^2+^ from intracellular Ca^2+^ store through inositol 1, 4, 5-triphosphate (IP_3_) receptors, and ryanodine receptors was also involved in the occurrence of SCOs [18,32,47]. In the current study, we demonstrated that BmK NSPK increased the SCO frequency and amplitude and depolarized membrane with low apparent nanomolar affinity in SCN cultures, consistent with its high affinity on the outward potassium channels. Therefore, it is reasonable to conclude that BmK NSPK-enhanced SCO frequency and amplitude is originated from its inhibition of outward K^+^ currents in SCN cultures. BmK NSPK-enhanced SCO frequency and amplitude is likely stepped from its ability to produce membrane depolarization that enhances rhythmic glutamate release in the pre-synapse that triggers Ca^2+^ release through metabotropic glutamate receptor subtype 5 (mGluR5)-coupled phospholipase C/IP_3_ receptor pathways [18]. Slight membrane depolarization also can facilitate the action potential generation that may contribute to increased SCO frequency [48], as SCO was dependent on the action potential firing [46]. BmK NSPK-induced membrane depolarization is likely resulted from its inhibition of I_A_ and I_D_ currents. This is consistent with 4-aminopyridine, a transient K_v_ channel blocker, and tetraethylammonium, a delayed-rectifier K_v_ channel blocker, response on the membrane potential [49,50].

Neuronal activity regulates intracellular Ca^2+^ dynamics, and activity-dependent calcium signaling regulates neurite extension and dendritic branching [51]. In the current study, we demonstrated that BmK NSPK, a short-chain scorpion peptide, enhanced neurite outgrowth in a nonmonotonic manner. This nonmonotonic stimulation on the neurite outgrowth is consistent with activity-dependent neurite outgrowth by activation of VGSCs [52], activation of ryanodine receptors [32], or suppression of K_v_1 channels [18]. We previously have reported a long-chain α-scorpion toxin (BmK NSP) that directly activated VGSCs by delaying inactivation of VGSCs [7]. BmK NSP also promoted neurite outgrowth that was suppressed by the inhibitors of L-type Ca^2+^ channels, N-methyl-D-aspartic acid receptors, and Na^+^-Ca^2+^ exchangers [7]. Although the currently reported short-chain scorpion toxin, BmK NSPK, also promoted the neurite outgrowth, the molecular mechanism was distinct from that of BmK NSP. BmK NSPK had little effect on VGSCs. The effect of BmK NSPK on neurite outgrowth likely resulted from its inhibition of K_v_ channels. Although both Bmk NSP and BmK NSPK affected the intracellular Ca^2+^ signaling, the phenotypes and molecular mechanisms were distinct. BmK NSP produced sustained intracellular Ca^2+^ elevation through L-type Ca^2+^ channels, N-methyl-D-aspartic acid receptors, and reverse mode of Na^+^–Ca^2+^ exchangers that were a consequence of activation of VGSCs [7,9]. However, BmK NSPK increased SCO amplitude and frequency. The effect of BmK NSPK on SCOs was similar to that of K_v_1 and K_v_3.1 channel blockers, which augmented SCO frequency and amplitude by enhancing Ca^2+^ release through mGluR5-coupled phospholipase C/IP_3_ receptor pathways [18].

An interesting finding was that BmK NSPK exposure stimulated NGF release in SCN cultures. NGF release can be augmented by depolarization, and depolarization augments Ca^2+^ signaling [53,54]. Therefore, it is likely that BmK NSPK-augmented release of NGF is triggered by the inhibition of outward potassium channels that leads to the membrane depolarization and augmented Ca^2+^ signaling. NGF is essential for proper development, patterning, and maintenance of nervous systems. NGF has been reported to enhance the neurite outgrowth in PC12 cells [38] and primary sensory neurons [39] through selective binding to TrkA receptors [55]. We demonstrated that GW 441756, a TrkA receptor inhibitor, eliminated BmK NSPK-induced neurite outgrowth, suggesting that the NGF/TrkA pathway was critical in the regulation of BmK NSPK-induced neurite outgrowth. Both NGF- and BmK NSPK-induced neurite outgrowth displayed a bell-shaped response that was also similar to the pattern of activity-dependent neurite outgrowth. Therefore, these data infer that activity-dependent neurite outgrowth is also likely through the NGF/TrkA pathway. Akt, the key molecules downstream of TrkA receptors, have been reported to be involved in survival, regeneration, and differentiation of neurons [40,56]. We demonstrated that BmK NSPK also stimulated the phosphorylation levels of Akt, further demonstrating the involvement of the NGF/TrkA signaling pathway in BmK NSPK-stimulated neurite outgrowth.

In summary, we purified a potent potassium channel blocker, designated to be BmK NSPK, from the scorpion *Buthus martensii* Karsch. We further demonstrated that BmK NSPK increased SCO frequency and amplitude, and stimulated neurite outgrowth in a nonmonotonic manner through NGF/TrkA receptor signaling pathway in SCNs. Potassium channels may represent molecular targets to modulate spinal cord regeneration and to develop the treatments of spinal cord injury.

## 4. Materials and Methods

### 4.1. Animal Care

All the animal protocols were approved by the Institutional Animal Care and Use Committee (#SYXK 2016-0011) of China Pharmaceutical University at 31 March, 2020. Efforts were made to reduce the number of experimental animals and to minimize animal suffering. C57BL/6 mice (18–22 g) were purchased from Qinglongshan Laboratory Animal Center (Nanjing, China). The animals were housed under standard environmental conditions (12/12 h light/dark cycle at 23 ± 2 °C). The chow and tap water were available ad libitum.

### 4.2. Materials

BmK venom was purchased from a domesticated scorpion farm (Kaifeng, China), where it was collected by electrical stimulation. Sephadex G-50 and CM-Sephadex C-50 were purchased from Pharmacia Fine Chemicals (Uppsala, Sweden). Acetonitrile (HPLC grade) was from Tedia (Cincinnati, OH, USA). Dialysis membrane (500 Da cut off) was a product of Minnesota Mining and Manufacturing (St. Paul, MN, USA). Trypsin, L-glutamine, fetal bovine serum, Neurobasal medium, Hoechst 33,342, and anti-MAP2 primary antibody were obtained from Life Technology (Grand Island, NY, USA). Primary antibodies against Akt, phosphorylated (p)-Akt, were obtained from Cell Signaling Technology (Danvers, MA, USA). Secondary antibodies and NewBlot Nitro Stripping Buffer were from LI-COR Biotechnology (Lincoln, NE, USA). Trifluoroacetic acid, cytosine arabinoside, poly-L-lysine, N-2-hydroxyethylpiperazine-N-2-ethane sulfonic acid (HEPES), GW 441756, and all inorganic salts were obtained from Sigma-Aldrich (St. Louis, MO, USA). Tetrodotoxin was purchased from Tocris Bioscience (Ellisville, MO, USA). The Ca^2+^-specific fluorescent dye Fluo-4/AM was obtained from AAT Bioquest (Sunnyvale, CA, USA). NGF ELISA kit was purchased from Kete Biological Technology Co., Ltd. (Nanjing, China).

### 4.3. BmK NSPK Purification

The scorpion venom was dissolved in ddH_2_O and loaded to a Sephadex G-50 column (35 × 700 mm) eluted with ddH_2_O. Four peaks (G-1 to G-4) were collected based on the absorbance at 280 nm. Fraction G-2 was loaded onto a pre-equilibrated CM-Sephadex C-50 cation ion-exchange column (26 × 500 mm) as described previously [21]. The fraction eluted with 0.5 M NaCl was dialyzed, lyophilized, and further separated in an Ultimate XB-C18 reverse-phase semipreparative column (9.6 × 250 mm, Welch Materials Inc., Shanghai, China) using an Agilent 1260 Infinity LC (Agilent Technologies, Santa Clara, CA, USA) high-performance liquid chromatography (HPLC) system. The column was eluted with different percentages of solvent A (0.1% Trifluoroacetic acid (TFA) in ddH_2_O) and solvent B (0.085% TFA, 70% acetonitrile in ddH_2_O) over time: from 0 to 5 min 19% B, 5–20 min, 28% B; 20–55 min, 48% B; 55–60 min, 19% B at a flow rate of 2 mL/min.

### 4.4. Mass Spectrometry

A Quattro micro triple quadrupole mass spectrometer (Waters, Milford, MA, USA) was used to determine the molecular weight of purified peptide by electrospray ionization (ESI) in a positive mode. The desolvation temperature was set at 300 °C, and the source temperature was 110 °C. The capillary voltage was 3.0 kV, with cone voltage at 30 V. Nitrogen was used as cone gas and desolvation gas. Peptide was dissolved in a mixture of acetonitrile: ddH_2_O at a ratio of 1:1 containing 0.1% formic acid.

### 4.5. Edman Degradation

The amino acid sequence of BmK NSPK was determined by Edman degradation on a Protein Sequencer, model PPSQ-33A (Shimadzu Co., Kyoto, Japan) as described previously [21].

### 4.6. Sequence Alignment and Molecular Modeling

The amino acid sequences of BmK NSPK and toxins from the Swiss-Prot database were compared and aligned using ClustalX software (v2.0, University College Dublin, Dublin, Ireland). Three-dimensional structure modeling of BmK NSPK was carried out by homology modeling using SWISS-MODEL workspace [57] using BmKTX (PDB code: 1BKT) [23] as a template.

### 4.7. Primary Culture of Spinal Cord Neurons (SCNs)

SCN cultures were performed as described previously [7]. The dissociated neurons were planted into poly-L-lysine-coated 35 mm Petri dishes, 96-well plates at densities of 2 × 10^3^ cells/dish for patch clamp, 2.25 × 10^5^ and 2 × 10^3^ cells/well for Ca^2+^ imaging and neurite outgrowth experiments, respectively. Cells at 6–8 DIVs (days in vitro) were used to measure K^+^ and Na^+^ currents, membrane potential, or intracellular Ca^2+^ concentration ([Ca^2+^]_i_).

### 4.8. Intracellular Calcium Concentration Measurement

The intracellular Ca^2+^ concentration [Ca^2+^]_i_ of SCNs was measured using FLIPR^Tetra®^ system (Molecular Devices, Sunnyvale, CA, USA) as described previously [32].

### 4.9. Whole-Cell Patch-Clamp Recording

Current-clamp recordings can provide useful information about the contribution of a channel to membrane potential. Membrane potentials, Na^+^ and K^+^ currents in SCNs (6–8 DIVs), were recorded using whole-cell patch clamp using an EPC-10 amplifier and PatchMaster software (HEKA, Pfalz, Germany). Pipettes were pulled from 1.5 mm capillary tubing using a P-1000 puller (Sutter Instrument, Novato, CA, USA). The pipette resistance was 2–4 MΩ.

For current-clamp recordings, to record changes in membrane potential, patch pipettes were filled with intracellular solution containing the following (in mM): KCl 140, MgCl_2_ 5, CaCl_2_ 2.5, EGTA 5, ATP 4, GTP 0.3, and HEPES 10 (pH adjusted to 7.3 with KOH). The external solution contained the following (in mM): NaCl 140, MgCl_2_ 1, KCl 5, CaCl_2_ 2, glucose 10, and HEPES 10 (pH adjusted to 7.3 with NaOH). The membrane potentials were recorded by a 0 pA input current with a duration of 1 s. For voltage-clamp recordings, to record potassium currents, patch pipettes were filled with an internal solution containing (in mM): KCl 140, CaCl_2_ 1, MgCl_2_ 2.5, HEPES 10, EGTA 11, and ATP 4, and pH was adjusted to 7.2 with KOH. Cells were bathed in an external solution containing the following (in mM): choline chloride 150, KCl 5, MgCl_2_ 1, HEPES 10, D-glucose 10, and NMDG 2, and pH was adjusted to 7.4 with KOH. To record sodium currents, patch pipettes were filled with an internal solution containing the following (in mM): CsF 135, HEPES 5, and NaCl 10, and pH was adjusted to 7.2 with CsOH. Cells were bathed in external solution containing the following (in amM): NaCl 30, MgCl_2_ 1, CaCl_2_ 1.8, CsCl 5, D-glucose 25, KCl 5, HEPES 5, TEACl 90, and KCl 5, and pH was adjusted to 7.4 with NaOH. Capacitance and series resistance were compensated using computer-controlled circuitry of the amplifier. The outward potassium currents (I_K_), which contain both transient (I_A_) and sustained delayed-rectifier (I_D_) components, were elicited by a series of depolarizing pulses from −110 mV to the potentials ranged from −60 to +80 mV in a 10 mV increment. To record the I_D_ currents, voltages were stepped to the potentials ranged from −60 to +80 mV in a 10 mV increment from a 200 ms pre-depolarization potential of −40 mV that inactivated I_A_ currents. I_A_ currents were obtained by subtracting I_D_ currents from I_K_ currents. Na^+^ currents were elicited by depolarization steps from a holding potential of −100 mV to +45 mV in a 5 mV increment. Data were collected and analyzed using PatchMaster (HEKA Electronics, Pfalz, Germany) and SigmaPlot (version 10.3, Sigma-Aldrich). Concentration–response curves were fitted using the Hill equation: I_nor_= C + A/[1 + ([BmK NSPK]/EC_50_)*^p^*] [9], where [BmK NSPK] is the BmK NSPK concentration, C is the offset, the A values were always held to the values obtained under control conditions, I_nor_ is the normalized peak current (I_normalize_), EC_50_ is the half-maximal effective concentration, and p is the Hill slope [58].

### 4.10. Immunocytochemistry

Immunocytochemistry was performed as described previously [7]. After fixation, permeabilization, and blocking, neurons were incubated with primary antibody against MAP2 (1:1000) overnight at 4 °C and Alexa Fluor 488-conjugated goat anti-rabbit secondary antibody (1:1000) for 1 h at room temperature (RT), respectively. Hoechst 33,342 (1.6 μg/mL) was added into each well and incubated for 20 min to stain the nuclei. The images were captured by an eclipse inverted fluorescence microscope (Nikon, Tokyo, Japan). Neurites were manually tracked using ImageJ software (FIJI, version 1.51 g, NIH, Bethesda, MD, USA). Only the neurons with total neurite length ≥50 μm were used for quantification. The experiment was performed double blindly.

### 4.11. Enzyme-Linked Immunosorbent Assay (ELISA)

The NGF levels in the culture medium were determined by enzyme-linked immunosorbent assay (ELISA) kits according to the manufacturer’s instructions.

### 4.12. Western Blot

Equal amounts (30 μg) of cell lysates were loaded onto a 10% SDS-PAGE gel and transferred to a nitrocellulose membrane by electroblotting. After blocking, the blots were incubated with primary antibodies against p-Akt (1:1000) overnight at 4 °C and then incubated with the IRDye (680 RD or 800 CW)-labeled secondary antibodies (1:10,000) for 1 h at RT. The membranes were scanned and densitometry was quantified using LI-COR Odyssey Infrared Imaging System and its application software (version 2.1, LI-COR Biotechnology). The membranes were stripped with stripping buffer (LI-COR Biotechnology) and reblotted for analysis of the expression of Akt. Beta-tubulin (1:10,000) was used as a loading control.

### 4.13. Data Analysis

All data were presented as mean ± SEM. Graphing and data analysis were performed using GraphPad Prism software (Version 6.0, San Diego, CA, USA). Concentration–response curves were fitted by a nonlinear logistic equation. The IC_50_ values and 95% confidence intervals (CI) were determined by nonlinear regression using GraphPad Prism software. Statistical significance between different groups was calculated using an ANOVA and, where appropriate, a Dunnett′s multiple comparison test. A *p*-value below 0.05 was considered statistically significant.

## Data Availability

Data sharing not applicable. No new data were created or analyzed in this study. Data sharing is not applicable to this article.
